# Undiagnosed Autism Spectrum Disorder in a Child With Chronic Pain: A Case Report

**DOI:** 10.7759/cureus.56946

**Published:** 2024-03-26

**Authors:** Maelle Byl, Anne-Charlotte Morere, Christine Fonteyne

**Affiliations:** 1 Pediatrics, Hopital Universitaire de Bruxelles, Brussels, BEL; 2 Psychiatry, Hopital Universitaire de Bruxelles, Brussels, BEL

**Keywords:** psychiatric comorbidity, acute pain, neurodevelopmental disorders, chronic pain management, autism spectrum disorder (asd)

## Abstract

The literature acknowledges the presence of psychiatric comorbidities in pediatric chronic pain populations. Few studies have focused on comorbidity with autism spectrum disorders. We describe the case of a 10-year-old patient at the onset of his care by the chronic pain team. This boy had been experiencing refractory multifocal chronic pain for three years and had undergone multiple medical examinations that had not identified the cause of the pain or provided sufficient pain relief. During our consultations, the behavioral peculiarities (averted gaze, inhibition), the atypical description of this boy's pain (pain in the hair), and sensory peculiarities (intolerance to noise) led us to suspect an autism spectrum disorder. A multidisciplinary approach, including a thorough developmental history and evaluation by an autism resource center, confirmed this suspicion. The diagnosis of an underlying autism spectrum disorder allowed us to guide our management by integrating the specific sensory aspects of this boy. Concurrently, we facilitated the family's better understanding of the young boy's issues and addressed his social and communication difficulties. Through multidisciplinary care and the integration of these various aspects, our patient's clinical situation improved. Multidisciplinary management is essential in chronic pain teams.

## Introduction

The American Pain Society defines chronic pain as pain that extends beyond the expected period of healing and does not serve the alarm function of nociception [1]. It can be primary or secondary to a chronic illness [2]. It represents a common and often underestimated issue in pediatrics [2], with an estimated prevalence between 15% and 30% [3]. In children, it can lead to school absenteeism [4], functional limitations, a decrease in quality of life, and long-term persistence of chronic pain and mental health issues in adulthood [5,6]. Pediatric chronic pain is a complex syndrome resulting from the integration of biological processes with psychological, social, and cultural factors within a developmental trajectory [1].

The literature acknowledges the presence of psychiatric comorbidities in pediatric and adult chronic pain populations, such as anxiety, depressive moods, and behavioral problems [7,8]. However, to our knowledge, few studies have addressed the question of neurodevelopmental disorders in these populations [9]. According to the Diagnostic and Statistical Manual of Mental Disorders, Fifth Edition (DSM-V) [10], neurodevelopmental disorders are defined as a set of conditions that begin in early childhood. They are characterized by developmental deficits leading to impaired personal, social, academic, or occupational functioning [10]. This includes autism spectrum disorders characterized by persistent deficits in social interaction and communication, as well as repetitive and restricted behaviors and interests [10].

Within the pediatric chronic pain consultation at the Hôpital Universitaire des Enfants Reine Fabiola (Hôpital Universitaire de Bruxelles), we offer patients and their families multidisciplinary care using a biopsychosocial approach, systematically including somatic, psychological, social, and environmental evaluations, and, if necessary, pediatric psychiatric assessments. We describe the clinical case of a patient with multifocal chronic pain without an identified underlying cause and with an undiagnosed autism spectrum disorder before being managed by our team.

## Case presentation

A 10-year-old boy was referred to the pediatric chronic pain clinic for complaints of pain throughout his body, including headaches, abdominal pain, and musculoskeletal pain that had been ongoing for several years. The patient and his family explained that the pains began around the age of seven and a half with no medical or surgical history prior to the onset of pain. Over a few weeks, he developed painful complaints at multiple locations (thoracic, abdominal, pelvic, muscular, and headaches). There were no identified triggering factors. The pains were frequent and almost daily, presenting as brief episodes (lasting from a few seconds to five minutes). The child reported no other associated symptoms. However, he described that sometimes the pain was so intense that he could not move and/or would drop whatever he was holding. The pain did not wake him up at night.

Cardiological (electrocardiogram) and neurological (standard and prolonged electroencephalogram, brain MRI) medical assessments revealed no abnormalities. He underwent abdominal ultrasound and a contrast-enhanced abdomen, which were normal except for slight fecal impaction requiring no specific intervention. Stool analysis and lactose tolerance test also turned out normal. Throughout this three-year period, the management of his pain relied on occasional analgesic treatment (ibuprofen) with limited effectiveness.

**Figure 1:**
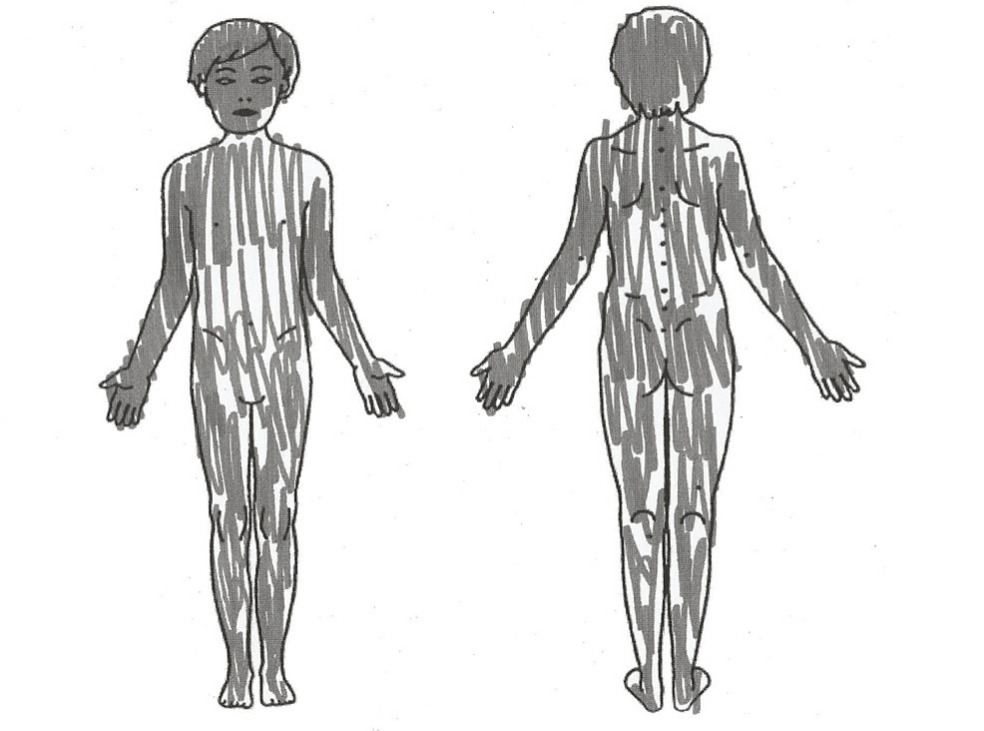
Localizations of the painful areas of the body The patient was asked to color the painful areas of his body. He colored all areas of his body, indicating that they were very painful.

At the age of 10, because of the persistence and lack of improvement in his pain, the patient was referred to the pediatric chronic pain clinic. Joint interviews involving a pediatrician, child psychiatrist, and nurse were conducted with the patient and his family. During these interviews, the healthcare team was surprised by the atypical description of the pain presented by the child. The patient described the same pains as before but also reported unpleasant sensations that he found difficult to specify. He localized his pains throughout his entire body, including his hair, and reported feeling his blood flowing in his vessels (Figure 1).

He reported brief sensations of electric shock throughout his body, not hindering his daily functioning. He did not experience painful burning or cold sensations and had no numbness. He described holocranial headaches, sometimes present from the morning, without nausea or vomiting. He mentioned discomfort in light and noise. He occasionally missed school when experiencing headaches. However, his academic performance was good. There were three school changes between preschool and primary school because of moving. The parents also reported a history of school bullying and anxious behavior in the child.

The patient comes from a Colombian family with no health issues. There is no family history of chronic pain. In the context of the atypical nature of the patient's pain complaints and his withdrawn and elusive demeanor during consultations, the team suspected autism spectrum disorder. A comprehensive developmental history was conducted by the child psychiatrist. The mother reports a stressful pregnancy with numerous episodes of vomiting and dehydration. The patient was delivered vaginally at 38 weeks gestation, weighing 2.1 kg. There were no neonatal incidents. The patient has had difficulties with falling asleep since infancy. He has never shown interest in a comfort object or pacifier. In terms of psychomotor development, there was early language acquisition (first words at eight months and full sentences at two years), with a preference for precise language, and walking was achieved at 14 months. In terms of sensory issues, the parents report sensitivity to noise (intolerance to the noise of hairdryers, vacuum cleaners, or the clinking of utensils against plates, resulting in him covering his ears). Behaviorally, the parents describe a child who is not very interested in interacting with other children, rather isolated, with limited emotional facial expressions. There were no reports of flapping or tiptoe walking, but occasional spinning in circles was observed. The parents described him as inhibited and withdrawn. He has never sought the company of his peers, although he reportedly has a friend at school. The child also has restricted interests, particularly in drawing (with great precision, sometimes using it to interact with others) and playing the piano (absolute pitch). Despite the patient's atypicalities, the diagnosis of autism spectrum disorder had never been addressed previously, neither by the school nor by healthcare providers who had been in contact with him and his family before. Based on the developmental history and the patient's atypical complaints, the healthcare team proposed to the family a referral to an autism resource center parallel to pain management. The autism resource center evaluation confirmed the diagnosis of autism spectrum disorder and highlighted difficulties, especially in communication and socialization based on the Autism Diagnostic Interview-Revised (ADI-R) and the Autism Diagnostic Observation Schedule (ADOS). Intellectual assessment (Wechsler Intelligence Scale for Children®-V) showed a heterogeneous but average intellectual profile (Table 1). The patient has recently joined a social skills and language pragmatics group.

**Table 1:** Composite score summary of the Wechsler Intelligence Scale for Children®-Fifth Edition (Wechsler Intelligence Scale for Children®-V)

Composite	Sum of scaled scores
Verbal comprehension	89
Visual-spatial	122
Fluid reasoning	109
Working memory	112
Processing speed	111
Full-scale intellectual quotient	Not calculated because of heterogeneous profile

The chronic pain team supported the family and the child in a multimodal approach, including psychoeducation about chronic pain (for both parents and the child) and awareness of psychocorporal techniques. The comprehensive (biopsychosocial) care and integration of various perspectives gradually led to an improvement in the chronic pain that the young boy had been experiencing for three years. Physical methods (massages) proved effective in alleviating his pain. During consultations, sleep difficulties (difficulty falling asleep and early awakenings) were highlighted and addressed. Sleep assessment using a sleep diary and the initiation of extended-release melatonin treatment helped improve the patient's sleep. The young boy was also guided toward resuming regular physical activity (cycling). The combined approach targeting various biological aspects (physical pain management methods, increased physical activity), psychological aspects (improved sleep, addressing issues of anxiety, school difficulties, and bullying), and social aspects (education on pain and the social functioning peculiarities associated with autism disorder traits) gradually led to a reduction in the intensity of pain peaks, decreased pain complaints, and an overall improvement in the patient's functioning. Currently, the patient continues to attend school, and follow-up appointments have been spaced out because of the significant improvement in pain.

## Discussion

This article describes the case of a 10-year-old boy presenting with multifocal chronic pain, with no identified cause and resistance to analgesic treatments (ibuprofen). An autism spectrum disorder is diagnosed during the management of chronic pain, three years after the onset of symptoms. The identification of his specific characteristics and family education regarding his autism spectrum disorder integrated into the chronic pain management by the healthcare team led to an improvement in the intensity and frequency of painful episodes. Other authors [11,12] have reported similar situations of children with refractory chronic pain, where a subsequent diagnosis of autism spectrum disorder improved the situation. In each of these situations [11,12], as in ours, in-depth developmental history allowed for the identification of autistic comorbidity in patients. Indeed, all these patients exhibit a good level of intellectual functioning, contributing to the delayed diagnosis of autism spectrum disorder. It appears crucial to have multidisciplinary teams when evaluating children with chronic painful complaints and/or atypical painful complaints. Multidisciplinarity seems to enhance not only the initial patient assessment but also pain management strategies.

In a study conducted on a pediatric population with chronic pain, Lipsker et al. [13] screened for autistic traits using the Social Responsiveness Scale. They highlighted a prevalence of 13.7% of patients with autistic traits (20 patients out of 146). While the Social Responsiveness Scale is not a diagnostic tool, and the actual prevalence is likely lower, this comorbidity appears to be real and warrants attention. Despite several descriptions of clinical situations similar to ours, we, such as our colleagues [11], note a lack of literature and current knowledge.

It is possible that patients with autism spectrum disorder are more prone to sensory stimuli that can be perceived as painful. A study involving 973 adult autistic patients shows that 21% of them exhibit central sensitization syndrome [14]. Our young patient reports pain in his hair or the sensation of blood flowing in his veins. Several testimonials describe sensory perceptions of this kind of experience as painful, as reported by Grandin [15]. She mentions sensory perception abnormalities related to touch, hearing, and smell that can cause pain [15]. These perception abnormalities are also included in the DSM-V [10]. The debate does not end there as it also concerns the experience of acute pain by autistic patients. Several studies have assessed the perception of acute pain in autistic patients [16,17] and suggest that their perception of pain is normal but expressed differently. In their literature review, Ruelle-Le Glaunec et al. [18] describe multiple studies on populations with autism spectrum disorders, reporting either hypersensitivity, hyposensitivity, or normal sensitivity to acute pain. This demonstrates the lack of consensus regarding the issue of painful perception in patients with autism spectrum disorders. The DSM-V [10] also includes hypersensitivity and hyposensitivity in its definition. While many authors have focused on the question of acute pain [16-18], very few delve into the issue of chronic pain in patients with autism spectrum disorders. Patients with chronic pain are regularly excluded from studies on painful perception [16,17]. Therefore, we face a population potentially more susceptible to sensory stimuli perceived as painful and/or chronic pain but that we struggle to identify. The underlying diagnosis of autism spectrum disorder helps improve our understanding, which is of the patient and the family regarding chronic painful symptoms. It also opens other therapeutic avenues such as cognitive-behavioral therapy [12]. Autistic patients are known for their lack of cognitive flexibility [19]. Cognitive-behavioral therapy could theoretically enhance the ability to distract from pain and thus seems to be a therapy of choice. To our knowledge, however, no study has evaluated the effectiveness of this therapy in these patients.

## Conclusions

Among children and adolescents consulting for a chronic pain syndrome, some of them present a neurodevelopmental disorder such as autism spectrum disorder that has not been previously diagnosed. This emphasizes the importance of the multidisciplinarity of the medical team caring for pediatric patients with chronic pain. Multidisciplinarity allows for the assessment of the somatic, sensory, and developmental specificities of the patient. It helps identify and manage developmental comorbidities in the patient. The child psychiatrist contributes their expertise to both the assessment of patients and the implementation of management strategies. However, this work informs us that the subject is still relatively unknown, and healthcare professionals are not sufficiently aware of these aspects.

## References

[1] American Pain Society (2024). Assessment and management of children with chronic pain, a position statement from the American Pain Society. https://da7648.approby.com/m/3063bf5632bf22e3.pdf.

[2] Friedrichsdorf SJ, Giordano J, Desai Dakoji K, Warmuth A, Daughtry C, Schulz CA (2016). Chronic pain in children and adolescents: diagnosis and treatment of primary pain disorders in head, abdomen, muscles and joints. Children (Basel).

[3] King S, Chambers CT, Huguet A, MacNevin RC, McGrath PJ, Parker L, MacDonald AJ (2011). The epidemiology of chronic pain in children and adolescents revisited: a systematic review. Pain.

[4] Norton J, Southon N (2021). Exploring the prevalence of pediatric chronic pain and school absenteeism for therapists working in schools: a systematic review with meta-analysis. Phys Occup Ther Pediatr.

[5] Shelby GD, Shirkey KC, Sherman AL (2013). Functional abdominal pain in childhood and long-term vulnerability to anxiety disorders. Pediatrics.

[6] Horst S, Shelby G, Anderson J (2014). Predicting persistence of functional abdominal pain from childhood into young adulthood. Clin Gastroenterol Hepatol.

[7] Coffelt TA, Bauer BD, Carroll AE (2013). Inpatient characteristics of the child admitted with chronic pain. Pediatrics.

[8] Tegethoff M, Belardi A, Stalujanis E, Meinlschmidt G (2015). Comorbidity of mental disorders and chronic pain: chronology of onset in adolescents of a national representative cohort. J Pain.

[9] Vinall J, Pavlova M, Asmundson GJ, Rasic N, Noel M (2016). Mental health comorbidities in pediatric chronic pain: a narrative review of epidemiology, models, neurobiological mechanisms and treatment. Children (Basel).

[10] American Psychiatric Association (2015). Troubles neurodéveloppementaux. Manuel Diagnostique et Statistique des Troubles Mentaux (DSM-V).

[11] Wiwe Lipsker C, von Heijne M, Bölte S, Wicksell RK (2018). A case report and literature review of autism and attention deficit hyperactivity disorder in paediatric chronic pain. Acta Paediatr.

[12] Bursch B, Ingman K, Vitti L, Hyman P, Zeltzer LK (2004). Chronic pain in individuals with previously undiagnosed autistic spectrum disorders. J Pain.

[13] Lipsker CW, Bölte S, Hirvikoski T, Lekander M, Holmström L, Wicksell RK (2018). Prevalence of autism traits and attention-deficit hyperactivity disorder symptoms in a clinical sample of children and adolescents with chronic pain. J Pain Res.

[14] Grant S, Norton S, Weiland RF, Scheeren AM, Begeer S, Hoekstra RA (2022). Autism and chronic ill health: an observational study of symptoms and diagnoses of central sensitivity syndromes in autistic adults. Mol Autism.

[15] Grandin T (1992). An inside view of autism. High-Functioning Individuals with Autism.

[16] Vaughan S, McGlone F, Poole H, Moore DJ (2020). A quantitative sensory testing approach to pain in autism spectrum disorders. J Autism Dev Disord.

[17] Fründt O, Grashorn W, Schöttle D (2017). Quantitative sensory testing in adults with autism spectrum disorders. J Autism Dev Disord.

[18] Ruelle-Le Glaunec L, Inquimbert P, Hugel S, Schlichter R, Bossu JL (2021). [Nociception pain and autism]. Med Sci (Paris).

[19] Craig F, Margari F, Legrottaglie AR, Palumbi R, de Giambattista C, Margari L (2016). A review of executive function deficits in autism spectrum disorder and attention-deficit/hyperactivity disorder. Neuropsychiatr Dis Treat.

